# Giant Cell Arteritis: A Systematic Review of the Qualitative and Semiquantitative Methods to Assess Vasculitis with 18F-Fluorodeoxyglucose Positron Emission Tomography

**DOI:** 10.1155/2014/574248

**Published:** 2014-09-01

**Authors:** Cristina Puppo, Michela Massollo, Francesco Paparo, Dario Camellino, Arnoldo Piccardo, Mehrdad Shoushtari Zadeh Naseri, Giampiero Villavecchia, Gian Andrea Rollandi, Marco Amedeo Cimmino

**Affiliations:** ^1^Department of Radiology, Department of Diagnostic Imaging, E.O. Ospedali Galliera, Mura della Cappuccine 14, 16128 Genoa, Italy; ^2^Nuclear Medicine Unit, Department of Diagnostic Imaging, E.O. Ospedali Galliera, Mura della Cappuccine 14, 16128 Genoa, Italy; ^3^Research Laboratory and Academic Unit of Clinical Rheumatology, Department of Internal Medicine, University of Genoa, V.le Benedetto XV 6, 16132 Genoa, Italy

## Abstract

Giant cell arteritis (GCA) is the most common vasculitis affecting medium and large vessels. It shows a close clinical association with polymyalgia rheumatica (PMR), a musculoskeletal inflammatory disorder, which is clinically characterized by girdles pain and stiffness. 18F-Fluorodeoxyglucose (18F-FDG) positron emission tomography (PET) is an effective tool for the diagnosis, grading, and follow-up of patients affected by GCA involving the aorta and its proximal branches, but the lack of a standardized method for the assessment of vascular inflammation remains a critical issue, potentially leading to misclassification. In our systematic review, including 19 original articles for a total of 442 GCA patients (with or without PMR symptoms) and 535 healthy controls, we described the different qualitative, semiquantitative and combined methods that have been proposed throughout the literature for assessing the presence and grading the severity of GCA-related vascular inflammation on 18F-FDG PET scans, focusing on the diagnostic performance and examining their respective advantages and limitations. The majority of the included studies adopted qualitative methods of PET image analysis, which are less sensitive but more specific than semiquantitative ones. Among the semiquantitative approaches, the aortic-to-blood pool uptake ratio of the aortic arch seems to be the most accurate method.

## 1. Introduction 

Giant cell arteritis (GCA) is the most common vasculitis affecting medium and large vessels, with an incidence of 7–18 cases per 100,000 individuals and with women affected twice as often as men [1, 2]. GCA was initially described as temporal arteritis (Horton disease), but about 15–27% of patients have extracranial involvement, since the entire aorta and all its branches can be affected, including the carotid, subclavian, and iliac arteries [3–5]. Polymyalgia rheumatica (PMR) is an inflammatory disorder, two to three times more common than GCA and clinically characterized by girdles pain and stiffness. PMR can occur before and simultaneously with or develop after clinical manifestations of GCA [6–10].

Population-based studies have shown that PMR occurs in about 50% of patients with GCA, and approximately 15%–30% of PMR patients develop GCA [1, 11]. The presence of different clinical features common to both PMR and GCA (e.g., older age at onset with progressively increasing incidence rates after 50 years, similar sex ratio, substantial increase of acute-phase reactants, and rapid responsiveness to glucocorticoids) has suggested that they might be different manifestations of the same underlying process [1].

Although etiology, development mechanisms, and targets of inflammatory damage of both GCA and PMR have not been yet defined, there is increasing evidence that a combination of genetic, immunogenetic [12–17], and environmental factors may play a pivotal role [12, 18, 19].

Early detection of the involvement of thoracic aorta and its branches plays a fundamental role in patient management and treatment. Thoracic aortic aneurysms are more frequent in patients with GCA than in nonaffected people and tend to arise several years after the diagnosis, when other symptoms are less evident [5, 20].

Over the past recent years, 18F-FDG PET, computed tomography (CT) angiography, and magnetic resonance imaging (MRI) have revealed that extracranial involvement in GCA is more frequent than previously anticipated, occurring in 30–74% of patients [21–24].

18F-FDG PET is a functional imaging technique that has become an established tool in oncology [5] but it has demonstrated also a promising role in the field of inflammatory diseases [4, 5]. The main limitation of 18F-FDG PET/CT to become a reliable diagnostic tool is the lack of a standardized definition of vascular inflammation based on the intensity of the glucose analogue uptake. Several authors have proposed various 18F-FDG PET diagnostic criteria.

This systematic review is focused on the different qualitative and semiquantitative methods for diagnosis and grading of vascular inflammation in GCA patients (with or without associated PMR) by means of 18F-FDG PET. We also assessed the diagnostic performance and the clinical value of each method of evaluation.

Takayasu arteritis (TA) was not included in our analysis because, though sharing apparent similar FDG distributions with GCA, its target population, pathophysiology, evolution, and prognosis are not comparable with those of GCA.

## 2. Materials and Methods

### 2.1. Database Search

A systematic literature research was performed up to April 2014, with no time limits. PubMed and the Cochrane Library were searched for articles written in English that addressed the issue of 18F-FDG PET as a diagnostic tool in GCA with or without associated PMR. We used the MeSH query “giant cells arteritis” or “polymyalgia rheumatica” and “positron emission tomography.”

A first selection was based on the exclusion of review articles, meta-analyses, abstracts, editorials or letters, case reports, and studies investigating 3 or fewer patients because they failed to provide sufficient evidence-based data. In this first stage, two researchers independently reviewed titles and abstracts of all retrieved articles. Studies addressing 18F-FDG-PET as diagnostic tool in GCA/PMR were included, while articles related to other vasculitides were excluded.

**Figure 1:**
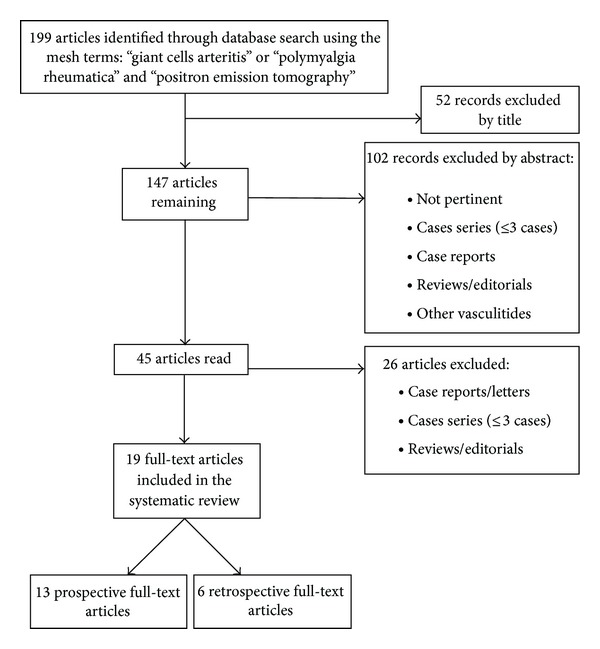
Flowchart of the review process.

At the second stage, the same two researchers independently assessed the full-text version of all articles that were found to be potentially eligible for inclusion, using the same inclusion and exclusion criteria as mentioned above (Figure 1). At both stages, disagreements between the two researchers were discussed and resolved by consensus.

## 3. Results

A total of 199 citations were found using the database search. Nineteen full-text articles, written from 1999 to 2014, were included. Of them, 13 were prospective and 6 retrospective studies. In order to assess vascular inflammation in GCA, with or without associated PMR, 10 studies used exclusively qualitative 18F-FDG uptake criteria, 6 used only semiquantitative criteria, and 3 adopted both qualitative and semiquantitative criteria (Table 1).

### 3.1. Qualitative Methods for the Assessment of 18F-FDG PET

**Figure 2:**
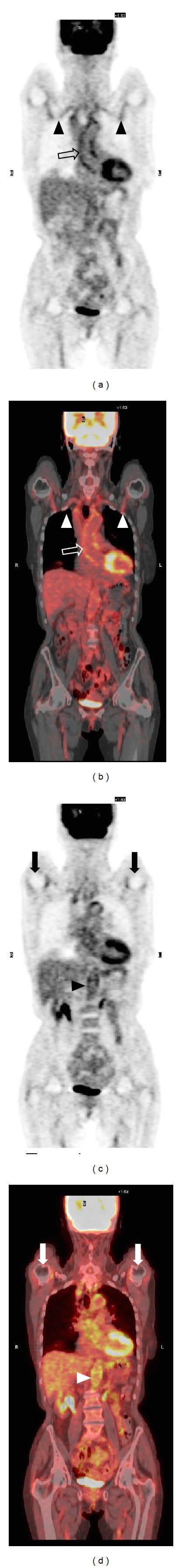
65-year-old female patient with 18F FDG PET-CT findings indicating the clinical association of polymyalgia rheumatica and giant cell arteritis. Coronal PET (a) and PET-CT (b) images demonstrate a significant tracer uptake of the walls of the ascending aorta, aortic arch (void arrows in (a) and (b)), and subclavian arteries (arrowheads in (a) and (b)). The second pair of coronal PET (c) and PET-CT (d) images demonstrate the inflammatory involvement of the abdominal aorta (arrowheads in (c) and (d)). A bilateral uptake of the tracer of the glenohumeral joints is also seen (solid arrows).

Ten studies [25–28, 30–32, 36, 38, 39] used exclusively qualitative methods of analysis to assess 18F-FDG uptake/accumulation within the walls of affected vessels in GCA/PMR patients (Figure 2).

Three of these articles [25, 26, 36] proposed a visual grading scale exclusively based on vascular 18F-FDG uptake and 5 studies [27, 31, 32, 38, 39] used a visual grading score based on the vessel-to-liver ratio. The remaining 2 studies [28, 30] defined each examination as positive or negative (i.e., abnormal versus normal), without specifying a positive threshold (Table 2).

In his first prospective study, Blockmans et al. [25] proposed a visual 4-point scale with scores ranging from 0 to 3, which was described as follows: a 0 score indicated no visualization of blood vessels; a score of 1 meant minimal 18F-FDG uptake, a score of 2 an increased 18F-FDG uptake, and a score of 3 18F-FDG a pronounced uptake. Blood vessels of the lower and upper limbs and the thoracic arteries were individually defined as positive for inflammatory involvement if the score was ≥2.

**Figure 3:**
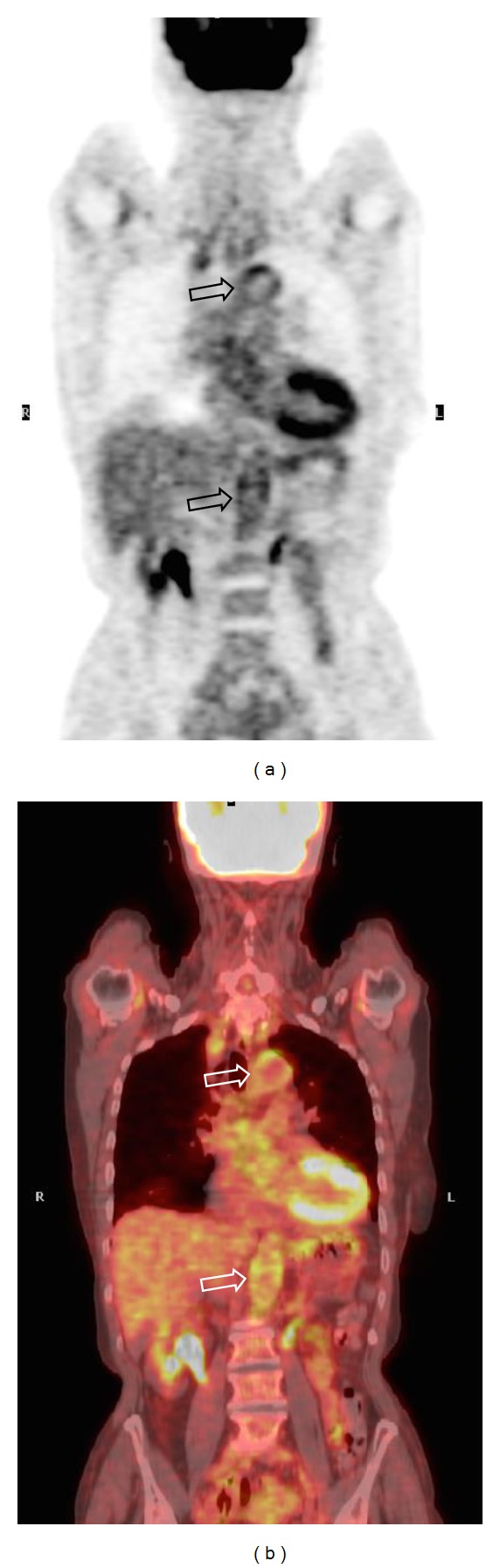
77-year-old female patient with clinical and PET-CT findings of giant cell arteritis. Coronal PET (a) and PET-CT (b) scans demonstrate the inflammatory involvement of aortic arch and abdominal aorta (void arrows), which is clearly appreciable by means of an immediate qualitative assessment of the images.

**Figure 4:**
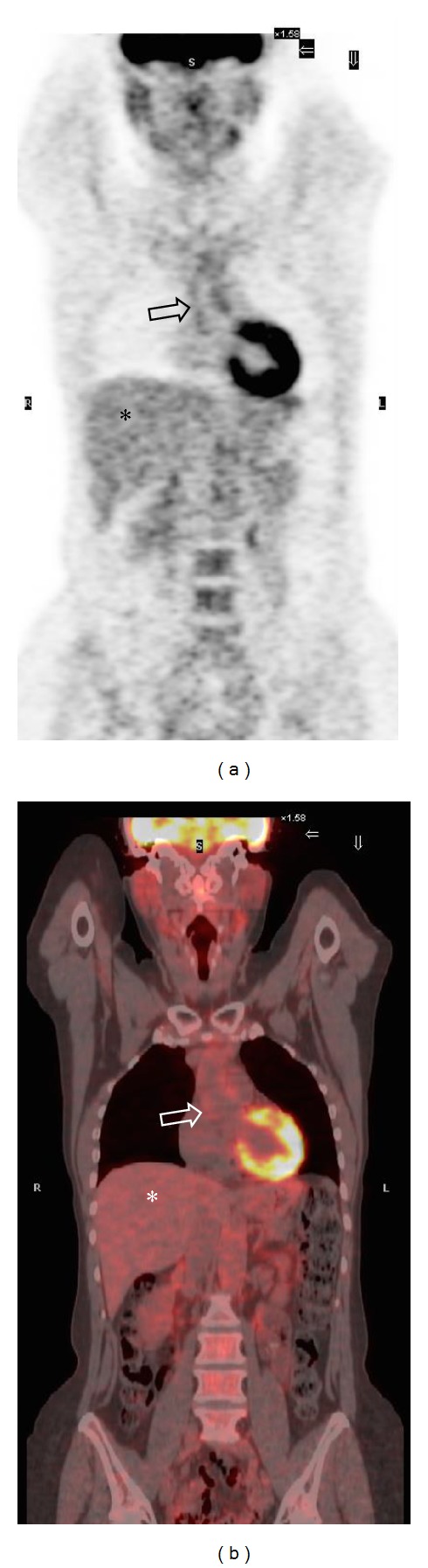
80-year-old male patient with clinical and PET-CT findings of giant cell arteritis. In this patient, coronal PET (a) and PET-CT (b) images demonstrate 18F FDG uptake of the walls of the ascending thoracic aorta (void arrows). The tracer uptake is similar to that of the liver parenchyma (asterisk), corresponding to grade 2 (significant vascular inflammation) according to the visual grading score proposed by Meller et al.

Brodmann et al. [30] examined 22 consecutive patients with clinical diagnosis of GCA confirmed by DUS: 18F-FDG PET scans were rated as negative or positive, without a definition of the criterion used (Figure 3). Meller et al. [27] proposed a visual grading scale, used by four other studies [31, 32, 38, 39], where large vessel 18F-FDG uptake was compared with that of the liver. According to this method, zero was defined as no uptake, 1 as uptake present but lower than liver uptake, 2 as uptake similar to liver uptake, and 3 as uptake higher than liver uptake (Figure 4). Three [27, 32, 39] out of these 5 studies concluded that a grade ≥2 for the thoracic aorta and a grade ≥1 in the other vascular regions were positive criteria for vasculitis. A smooth linear or long segmental pattern of 18F-FDG uptake in the aorta and its main branches, with an intensity higher than the liver uptake, was regarded as findings highly suggestive for GCA.

### 3.2. Semiquantitative Methods of Assessment of 18F-FDG PET

Six studies [21, 24, 33, 35, 40, 41] used a semiquantitative scoring method to evaluate vascular uptake (Table 3). Blockmans, in two prospective studies [21, 33], proposed a semiquantitative system that evaluates 18F-FDG uptake in 7 different vascular regions (thoracic aorta, abdominal aorta, subclavian arteries, axillary arteries, carotid arteries, iliac arteries, and femoral arteries) with the following grading: 0: no uptake; 1: minimal but not negligible uptake; 2: clearly increased uptake; and 3: very marked uptake. Based on this first evaluation, a total vascular score (TVS) was calculated, ranging from 0 (no vascular FDG uptake in any of the 7 vascular regions) to 21 (maximum vascular FDG uptake in all 7 locations). They found a mean TVS of 6 ± 0.2 at the time of diagnosis in 29 of 35 patients with GCA, with or without PMR. They also found a significantly lower mean TVS of 0.8 ± 1.7 in 31% of isolated PMR cases.

**Figure 5:**
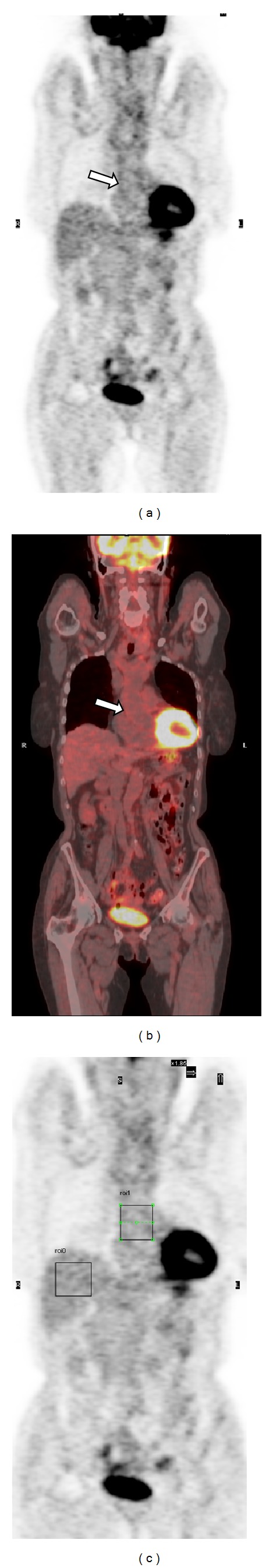
65-year-old female patient with polymyalgia rheumatica and suspected giant cell arteritis. An immediate qualitative visual assessment of the coronal PET-CT scan ((a) and (b)) led to the diagnosis of inflammatory involvement of the ascending thoracic aorta (white arrows). In this patient, the semiquantitative method of analysis proposed by Hautzel et al. was further applied (aorta-to-liver SUVmax ratio). Placing a ROI on the ascending thoracic aorta in the coronal PET image (c), a SUVmax of 1.6 was obtained. The SUVmax obtained by drawing the same ROI comprehensive on the liver (c) was 2.2, and the resulting aorta-to-liver SUVmax ratio was 0.7, below the cut-off value of 1 for diagnosing significant vascular inflammation. This is an example of discrepancy between qualitative and semiquantitative methods of image analysis.

Hautzel et al. [35] introduced a semiquantitative aorta-to-liver SUVmax ratio. In their prospective study they found an optimal relationship of sensitivity to specificity at an aorta-to-liver ratio of 1.0, even in patients with altered hepatic metabolism (Figure 5).

In their retrospective controlled study [41], Besson et al. compared three different semiquantitative approaches. The first method was derived from that described by Hautzel et al. [35], including two variants for normalizing the arterial activity to the liver uptake (i.e., highest SUVmax vascular/liver ratio and average SUVmax vascular/liver ratio) [35, 41]. The second approach was adapted from that described by Moosig et al. [29] and included two variants, where the arterial activity was normalized to the lung uptake (i.e., highest SUVmax vascular/lung ratio and average SUVmax vascular/lung ratio). The third approach had not been previously applied in GCA/PMR, but it was originally tested in atherosclerosis patients to evaluate arterial wall inflammation [42]. It was based on the arterial-to-blood pool uptake ratio and included two variants: in the first, the highest arterial SUVmax was normalized to the highest venous SUVmax; in the second, the average arterial SUVmax was normalized to the venous blood pool activity. The latter one was calculated by averaging the values obtained from eight ROIs drawn on axial 18F-FDG images in the right internal jugular vein.

### 3.3. Combined Qualitative and Semiquantitative Methods

Three studies [29, 34, 37] used a first qualitative analysis to diagnose or rule out the presence of vasculitis, performing a further semiquantitative assessment on vascular 18F-FDG uptake.

In the study of Moosig et al. [29], the visual examination of PET scans showed an increased tracer uptake in the aorta or in its major branches in 12 out of 13 included patients. For the semiquantitative assessment, 9 vascular areas were identified and sampled by placing different regions of interest. A peripheral region of the lung served to represent the background uptake, and vessel-to-lung SUVmax ratio was calculated. According to this method, Moosig et al. found that patients with active disease had significantly greater 18F-FDG uptake than control patients (mean ROI index of 1.58 versus 0.93).

Henes et al. [34] focused their analysis on the vessel showing the highest accumulation of the tracer at a first qualitative visual assessment. Further, they measured the maximum SUV on 6 locations of the selected vascular region. They found a mean SUVmax of 3.4 for all patients. The SUVmax was 3.9 in untreated patients versus 3.0 in patients under medical treatment.

Lehmann et al. [37] used two different diagnostic approaches for image assessment: a first visual analysis of vessel wall uptake compared to the background activity of liver, followed by a semiquantitative reevaluation, consisting of calculating the SUVmax in predefined regions of interest. They found that the SUVmax cut-off value of 1.78 is characterized by a high sensitivity (90% versus 65% of the qualitative visual assessment) and a low specificity (45% versus 80% of the qualitative visual assessment).

## 4. Discussion

The aim of our systematic review, which included a total of 442 cases of GCA patients, with or without PMR symptoms, and 535 controls, was to analyze the different qualitative and semiquantitative methods for assessing the presence and grading the severity of GCA-related vascular inflammation on 18F-FDG PET scans that have been proposed in the literature. We found the need for a standardized 18F-FDG PET interpretation in order to optimize the diagnostic performance of this imaging technique. Indeed, the lack of a standardized reading approach to defining vascular inflammation remains a critical issue and may lead to misclassification. Currently, the diagnosis of GCA is mainly based on clinical evaluation, laboratory findings, and temporal artery biopsy (TAB). The American College of Rheumatology diagnostic criteria for GCA do not include any imaging modality [43].

In patients with a distinctive clinical presentation, the diagnosis of GCA is not difficult and TAB is able to confirm the clinical suspicion. By contrast, a correct diagnosis may become challenging when symptoms are nonspecific, given the wide range of clinical manifestations of GCA [3, 5, 44]. Currently, TAB is still the diagnostic standard of reference [1, 2], but its routine clinical application is hampered by low sensitivity with a high false-negative rate (15%–40%) [45–49] and the concrete risk of underdetection [1, 9–12, 50]. Furthermore, the involvement of the thoracic aorta or its main branches, which is present in more than 45% of newly diagnosed patients with GCA, is associated with a negative TAB in 50% of reported cases [20, 51–54]. 18F-FDG PET is a noninvasive, whole-body technique that is able to detect vascular involvement in GCA patients, with or without PMR symptoms [25, 26]. Other imaging techniques (i.e., DUS, Doppler ultrasonography, CT, and MRI) have been proposed for the assessment of vascular inflammation in GCA patients. However, while they are able to demonstrate anatomical changes in the affected vessels (mural thickening, dilatation and aneurysms, and enhancement of perivascular connective tissue) if the inflammatory process is well established, they are not sensitive enough to diagnose early inflammatory changes which are potentially reversible [28]. Furthermore, patient follow-up and the assessment of response to medical treatment are not easy to perform on the basis of morphological information alone [27, 28].

DUS of temporal arteries has emerged as a useful alternative tool when temporal biopsy cannot be performed [52], but this technique is not able to demonstrate the involvement of thoracic vessels. 18F-FDG may be particularly helpful in patients with inconclusive TAB and/or DUS results [55, 56].

From the results of our review, we found that qualitative analysis of 18F-FDG uptake is the most widely adopted method for assessing the presence and grading the activity of GCA-related vascular inflammation on 18F-FDG PET scans (13 out of 19 original papers). Qualitative analysis has been used both to perform dichotomous assessment (i.e., confirm or rule out the presence of vascular inflammation) [28–31, 34, 35, 37, 39] and to grade the severity of vascular involvement according to ordinal scales, with the 18F-FDG uptake of the vessel wall being visually analyzed or compared with that of a reference structure [25–27, 32, 34, 36]. Among visual grading systems, the vessel-to-liver ratio is the most frequently used. With regard to diagnostic performance, a previous systematic meta-analysis compared different qualitative and semiquantitative methods for assessing vasculitis on 18F-FDG PET, reporting a pooled sensitivity and a specificity of 80% and 89%, respectively [51].

The strength of qualitative methods, in addition to being more immediate and less time-consuming than semiquantitative ones, lies in their high specificity and small number of false positives, while sensitivity ranges from 56% to 77%. Moreover, qualitative methods display high interobserver agreement and intraobserver reproducibility (90% and 93.3%, resp.) [32]. By contrast, most authors [24, 26, 28, 41, 42, 57–60] have reported that when qualitative methods are applied, the diagnostic discrimination between vasculitis and atherosclerosis may be a critical issue.

However, there is a general consensus that 18F-FDG vascular uptake in vasculitis should be higher than in atherosclerosis. For these reasons, mild vascular 18F-FDG uptake, lower than or equal to that of the liver (i.e., grades 1 and 2 according to scoring systems that use the liver as a reference structure), is not indicative of GCA inflammatory involvement. The site of tracer accumulation and its changes in response to medical treatment have also been considered to distinguish large vessel vasculitis from atherosclerosis [60].

A complementary contrast-enhanced CT acquired immediately after 18F-FDG PET/CT may provide additional information on the morphology of the affected vessels and the presence of calcifications within the arterial wall [34]. The metabolic information obtained from 18F-FDG PET, combined with the demonstration of wall enhancement and thickness assessed by contrast-enhanced CT, could constitute an effective approach to the evaluation of extracranial GCA, thereby enabling a better differentiation between GCA- and atherosclerosis-related 18F-FDG vascular uptake.

With regard to the semiquantitative methods, some authors have proposed the evaluation of arterial SUVmax [24, 34, 37], while others have normalized arterial SUVmax to the background activity, represented by the mean uptake value of a selected reference structure [29, 35, 41].

On comparing a SUVmax-based approach without normalization with visual qualitative analysis, Lehmann et al. [37] found high sensitivity (90% versus 65%) and low specificity (45% versus 80%) when a cut-off value of 1.78 was used. By contrast, Besson et al. [41] and Prieto-González et al. [24] found that SUVmax cut-off values obtained without normalization were population-specific and could not be applied to the general population.

From the results of our analysis, we observed that semiquantitative methods normalized to the background activity seem to outperform qualitative approaches and semiquantitative methods without normalization. We also noticed that the power of discrimination between GCA and control groups also depends on the anatomical structure chosen to represent the background uptake.

Besson et al. showed some limitations in the use of the liver as the background for normalization in GCA [41]. In particular, they found that the liver SUVmax differed significantly between patients and controls and that the liver SUVmax values in the GCA group were significantly higher than in the control group. Indeed, systemic inflammation affects liver metabolism and can influence the calculation of liver uptake values.

According to the results of our study, an interesting semiquantitative approach is the arterial-to-background ratio proposed by Moosig et al. [29]. These authors adopted a two-step procedure for assessing their patients. Initially, they applied a dichotomous qualitative method in order to identify positive cases; they then adopted a semiquantitative method, normalizing vascular 18F-FDG uptake to the lung. Indeed, the background activity of the lung displays low inter- and intrapatient variability owing to the low physiological uptake of 18F-FDG. Using this two-step procedure, Moosig et al. obtained maximal diagnostic performance values (i.e., both sensitivity and specificity of 100%) higher than those yielded by both qualitative and semiquantitative methods. Besson et al. [41] normalized vascular 18F-FDG uptake to the lung by means of a semiquantitative method similar to that of Moosig et al. and confirmed that the lung SUVmax values did not differ significantly between patients and controls. On the other hand, without performing the two-step combined procedure of Moosig et al. [29], Besson et al. [41] found lower values of sensitivity and specificity (81.8 and 72.7%, resp.).

Among all the semiquantitative methods for the assessment of 18F-FDG PET in GCA, the aortic-to-blood pool uptake ratio seems to outperform the other methods when liver and lung are used as reference structures for determining the background uptake.

This approach, tested by Rudd et al. in atherosclerosis to evaluate arterial wall inflammation, has proved to be robust and highly reproducible [57]. Rudd et al. normalized the arterial SUVmax values to blood pool activity, represented by the mean of 8 ROIs drawn in venous vessels (i.e., the inferior vena cava or the internal jugular vein). Besson et al. adapted this method in order to assess PET examinations of 11 GCA patients and 11 controls matched for age and sex and found high diagnostic sensitivity (81.8%) and specificity (91%).

## 5. Conclusion

In conclusion, 18F-FDG PET has been shown to have an important role in the diagnosis of extracranial vascular involvement in patients with GCA/PMR.

Qualitative methods are more specific than semiquantitative ones, but they have lower sensitivity. The aortic-to-blood pool uptake ratio is a promising semiquantitative method of analysis for the detection and grading of arterial inflammation. The normalization of the arterial wall uptake to the background activity of venous blood pool provides a good reference to assess vascular inflammation. Further prospective studies involving larger cohorts of GCA/PMR patients are required to better define the role of aortic-to-blood pool ratio as a reference method for the assessment of vasculitis in GCA patients.

## Figures and Tables

**Table 1:** Current state of scoring methods of 18F-FDG PET in giant cell arteritis.

Author (year)	Study design	Patients	Controls	Technique	Method of analysis	Description	Standard of reference	Diagnostic performance
Blockmans et al. (1999) [25]	Prospective	11	23	PET	Qualitative	Visual grading scale	ACR criteria + TAB	Not specified

Blockmans et al. (2000) [26]	Prospective	25	44	PET	Qualitative	Visual grading scale	Clinical symptoms + TAB	Thoracic vessels Sensitivity: 56% Specificity: 98% PPV: 93% NPV: 80% Legs Sensitivity: 64% Specificity: 77%

Meller et al. (2003) [27]	Prospective	15	Group 1: 38 Group 2: 40	PET and PET/CT	Qualitative	Visual grading scale	ACR criteria	Sensitivity: 73% Specificity: 100%

Bleeker-Rovers et al. (2003) [28]	Retrospective	22	—	PET	Qualitative	Positive/negative	ACR criteria	Sensitivity: 77% Specificity: 100% PPV: 100% NPV: 82%

Moosig et al. (2004) [29]	Prospective	13	6	PET	Qualitative and semiquantitative	Positive/negative and SUV vascular/lung ratio	PMR: exclusion of other causes of inflammation + Chuang and Healy criteria	Sensitivity: 100% Specificity: 100%

Brodmann et al. (2004) [30]	Prospective	22	—	PET	Qualitative	Positive/negative	ACR criteria + positive hypoechogenic halo on DUS	Not specified

Scheel et al. (2004) [31]	Prospective	8	—	PET and PET/CT	Qualitative	Positive/negative	Clinical symptoms	Not specified

Walter et al. (2005) [32]	Prospective	20	26	PET	Qualitative	Visual grading scale	ACR criteria	Sensitivity: 60% Specificity: 99.8% PPV: 99.7% NPV: 67.9% Accuracy: 78.6%

Blockmans et al. (2006) [21]	Prospective	35	—	PET	Semiquantitative	Visual grading scale	TAB	Not specified

Blockmans et al. (2007) [33]	Prospective	35	—	PET	Semiquantitative	Visual grading scale	PMR: clinical + negative TAB	Not specified

Henes et al. (2008) [34]	Prospective	13	—	PET/CT	Qualitative and semiquantitative	Positive/negative and highest SUVmax vascular	Clinical and diagnostic work-up (including DUS, MRI, CT, and TAB)	Sensitivity: 90% Specificity: 100%

Hautzel et al. (2008) [35]	Prospective	18	Group 1: 36 Group 2: 18	PET	Semiquantitative	Highest SUVmax aorta/liver ratio	ACR criteria or diagnostic work-up (including DUS, TAB, CT, and MRI)	Cut-off: 1.0 Sensitivity: 88.9% Specificity: 94.4%–95.1% Accuracy: 91.7%–93.2% PPV: 78.8%–88.9% NPV: 95.1%–97.7%

Both et al. (2008) [36]	Prospective	25	—	PET	Qualitative	Visual grading scale	Birmingham vasculitis activity score (BVAS.2)	Not specified

Lehmann et al. (2011) [37]	Retrospective	20	20	PET	Qualitative and semiquantitative	Positive/negative and highest SUVmax	Clinical (ACR) diagnosis confirmed by histology or MRI angiography	Visual grading Sensitivity: 65% Specificity: 80% SUVmax (cut-off 1.78) Sensitivity: 90% Specificity: 45%

Henes et al. (2011) [38]	Retrospective	10	—	PET/CT	Qualitative	Visual grading scale	Clinical symptoms	Not specified

Hooisma et al. (2012) [39]	Retrospective	62	242	PET/CT	Qualitative	Positive/negative	Clinical symptoms	Not specified

Yamashita et al. (2012) [40]	Retrospective	27	17	PET/CT	Semiquantitative	Visual grading scale	PMR: Chuang et al. and Healy's criteria; no clinical evidence of temporal arteritis	Not specified

Besson et al. (2013) [41]	Retrospective	33	11	PET/CT	Semiquantitative	*A*: highest SUVmax arterial/liver ratio	ACR criteria + TAB	Method C at aortic arch: cut-off value of 1.53
						*A*′: average SUVmax arterial/liver ratio		
						*B*: highest SUVmax arterial/lung ratio		Sensitivity: 81.8%
						*B*′: average SUVmax arterial/lung ratio		
						*C*: highest arterial SUVmax/highest venous SUVmax		Specificity: 91%
						*C*′: average arterial SUVmax/venous blood pool activity		

Prieto-González et al. (2014) [24]	Prospective	32	20	PET/CT	Semiquantitative	Highest SUVmax vascular/liver ratio	TAB	Any vascular territory (cut-off of 1.89) Sensitivity: 80% Specificity: 79% Epiaortic vessels (cut-off of 1.70) Sensitivity: 81% Specificity: 79% Aorta (cut-off 2.25) Sensitivity: 90% Specificity: 42% Aorta (cut-off 2.65) Sensitivity: 58% Specificity: 90%

**Table 2:** 18F-FDG PET and PET/CT qualitative diagnostic criteria.

Number of studies	Description
2	Positive/negative or normal/abnormal

3	Visual grading scale 0: none; 1: slight; 2: marked; 3: intense

5	Visual grading scale 0: no uptake; 1: lower than liver; 2: similar to liver; 3: more than liver

**Table 3:** 18F-FDG PET and PET/CT semiquantitative diagnostic criteria.

Number of studies	Description
3	Visual grading scale (0–3) and TVS
3	Highest SUVmax vascular/liver ratio
1	Average SUVmax vascular/liver ratio
1	Highest SUVmax vascular/lung ratio
1	Average SUVmax vascular/lung ratio
1	Highest SUVmax arterial/venous ratio
1	Average SUVmax arterial/venous ratio
